# Isoform specific gene expression analysis of *KRAS* in the prognosis of lung adenocarcinoma patients

**DOI:** 10.1186/s12859-018-2011-y

**Published:** 2018-02-19

**Authors:** In Seok Yang, Sangwoo Kim

**Affiliations:** Severance Biomedical Science Institute, Yonsei University College of Medicine, 50-1 Yonsei-ro, Seoul, 03722 South Korea

**Keywords:** Isoform specific expression, *KRAS* mutation, K-Ras4A, Lung adenocarcinoma, Survival

## Abstract

**Background:**

Aberrant mutations in *KRAS* play a critical role in tumor initiation and progression, and are a negative prognosis factor in lung adenocarcinoma (LUAD).

**Results:**

Using genomic analysis for K-Ras isoforms (K-Ras4A and K-Ras4B) and large-scale multi-omics data, we inspected the overall survival (OS) and disease-free survival (DFS) of LUAD patients based on the abundance of transcript variants by analyzing RNA expression and somatic mutation data from The Cancer Genome Atlas (*n* = 516). The expression of the minor transcript K-Ras4A and its proportion were positively correlated with the presence of *KRAS* mutations in LUAD. We found that both K-Ras4A abundance measures (expression and proportion) have a strong association with poor OS (*p* = 0.0149 and *p* = 3.18E-3, respectively) and DFS (*p* = 3.03E-4 and *p* = 0.0237, respectively), but only in patients harboring *KRAS* mutations. A Cox regression analysis showed significant results in groups with low expression (hazard ratio (HR) = 2.533, 95% confidence interval (CI) = 1.380−4.651, *p* = 2.72E-3) and low proportion (HR = 2.549, 95% CI = 1.387−4.684, *p* = 2.58E-3) of K-Ras4A.

**Conclusions:**

Based on the above results, we report the possible use of abundance measures for K-Ras4A for predicting the survival of LUAD patients with *KRAS* mutations.

**Electronic supplementary material:**

The online version of this article (10.1186/s12859-018-2011-y) contains supplementary material, which is available to authorized users.

## Background

*KRAS* mutations are present in approximately 30% of cases of lung cancer [1–4], in which amino acid alterations from Gly12 to Ala, Cys, Asp, and Val are most frequently detected. Aberrantly mutated *KRAS* has been shown to play a critical role in cancer initiation and maintenance by modulating oncogenic downstream effectors including Raf and PI3K, followed by the Raf/MEK/ERK and the PI3K/Akt pathways, respectively [5]. *KRAS* has also been shown to be a negative prognostic marker for lung cancer [3]. Mutual exclusiveness between *KRAS* and *EGFR* mutations [6] led to the three classifications of lung cancer groups: *KRAS* mutants, *EGFR* mutants, and *KRAS*/*EGFR* wild type [7]. However, no further stratifications have been available for lung cancer patients and *KRAS* mutations [7, 8]. Considering the prevalence of mutations in lung cancer and subsequent heterogeneous outcomes [2], we hypothesize that there are additional prognosis markers for patients with *KRAS* mutations, which may be possibly based on unseen traits of the gene.

*KRAS* amplification has been known to be frequently occurred in non-small cell lung cancer (NSCLC) [9, 10], of which the prevalence was reported to approximately 15%, revealing one of the common molecular alterations in NSCLC. Copy number gain of the gene has also been known to lead poor clinical outcome in NSCLC patients [11]. In addition, increased copy number of a gene may be closely related to its allelic imbalance. Indeed, a recent study revealed that *KRAS* showed imbalanced allelic expression in TCGA LUAD cancer type by comparing variant allele frequencies between DNA and RNA [12].

*KRAS* produces two splice variants (K-Ras4A and K-Ras4B) by alternative splicing, which are differentiated by alternative use of the last two exons [1]. Because the majority of *KRAS* mutations occur in the shared exon 2 (12th and 13th codons) and 3 (61st codon), both K-Ras4A and K-Ras4B isoforms are oncogenic. The isoforms also differ by the hypervariable region (HVR) sequences, where K-Ras4B contains a long polybasic stretch, while K-Ras4A has a short polybasic region with a palmitoylation site. As a result of their distinct genetic structures, their biological characteristics, such as plasma membrane binding, have been shown to be different [1, 13]. Generally, K-Ras4B has been shown to be the predominant form. However, frequent co-expression of the two isoforms has been found in multiple cancer types in a recent study [13]. Taken together, the expression pattern of the K-Ras isoforms can affect the cellular mechanisms of lung cancer, which may further influence the prognosis of the patients. Furthermore, a recent publication reported that K-Ras4A showed some structurally different characteristics compared to K-Ras4B as following: i) a more exposed nucleotide binding pocket in GDP-bound form; ii) different dynamic fluctuations in switch I and II regions; and iii) unstable autoinhibited state of HVR [14]. These results might imply some different roles in regulation of *KRAS* signaling between both K-Ras isoforms.

In light of high throughput transcriptome sequencing technology, such as RNA-seq [15] and related bioinformatics algorithms [16, 17], the traditional analysis of mRNA expression levels expression has been extended to the accurate quantification and structural determination of transcript variants. In addition, the availability of multi-omics cancer data from a large cohort, such as The Cancer Genome Atlas (TCGA) project [18], and convenient web platforms as demonstrated in our previous work [19], have enabled instant genome-level analyses. Inspection of the isoform-level traits of a gene provides deeper and more detailed insights to understand the biological characteristics of human cancers compared to previous gene-level analysis.

Here, we report the possible use of the expression pattern of K-Ras isoforms in the prediction of lung cancer survival, demonstrated by the statistical analysis of RNA-seq data from 516 patients with lung cancer adenocarcinoma (LUAD). We found that the abundance measurements (expression and proportion) for the K-Ras4A isoform are strongly associated with the presence of *KRAS* mutations as well as a positive prognosis for lung cancer patients harboring *KRAS* mutations. Multivariate analysis identified that the two measures of the isoform can be independent predictors.

## Methods

### Data acquisition and processing

We downloaded somatic mutation data files (level 2) and RNA sequencing (RNA-seq) data sets (level 3, RNA-seq v2 expression data) for LUAD from the TCGA Data Portal, which are currently stored in the Genomic Data Commons Legacy Archive [20] that is maintained by the National Cancer Institute (Additional file 1: Table S1).

For RNA-seq data sets, 516 tumor samples annotated as “primary solid tumor” were only included in this study. Transcripts per million (TPM) was used as a unit representing expression levels of genes and isoforms, which were calculated by multiplying the estimated fraction of transcripts made up by a given isoform or gene ranging from zero to one computed using RSEM [21]. An average TPM value was used if two or more expression levels of the gene or isoform were found for the same patient. A threshold of TPM > 10^− 6^ was applied to determine whether the gene or isoform was expressed or not as used in previous study [19]. Isoform proportions of a gene were calculated by dividing the TPM value of each isoform by the sum of TPM values of all isoforms.

Clinical information (Merge_Clinical, version 2016_01_28) for the LUAD patients was obtained from FireBrowse [22], which is maintained by the Broad Institute. The following metrics were extracted for comparisons of clinic-pathological characteristics, survival analysis, and Cox regression analysis: age, gender, smoking history, pathological stage, days to death, days to last follow-up, days to new tumor event after initial treatment, and vital status. Copy number alteration data (CopyNumber Gistic2, level4, version 2016_01_28) was also obtained from the FireBrowse [22], where the samples with *KRAS* amplification were determined with the threshold of 10%.

For comparison with the analysis results from LUAD, we also downloaded the same data sets and then prepared them as described above for the three cancer types (colon adenocarcinoma, COAD; pancreatic adenocarcinoma, PAAD; and rectal adenocarcinoma; READ) as shown in Additional file 1: Table S2.

### Identification of associations between the isoform and mutation state of *KRAS*

*KRAS* is one of the most frequently mutated genes in the LUAD cancer type [23]. Thus, the gene was targeted to examine for associations between the isoform and mutation state. After binning patient samples according to their orders sorted by expression levels or proportions for individual isoforms of the gene in each cancer type, a linear regression analysis was performed to calculate the R-squared (*r*^2^) and slope (*S*) with average expression level or proportion of each corresponding isoform and sum of the patients with *KRAS* mutations in each bin. If skewing of the line was found in the analysis, we recalculated *r*^2^ and *S* values after excluding outliers. We determined the presence or absence of an association with the *r*^2^ and *S* threshold of 0.6.

### Patient grouping

Patients of each cancer type were divided into high and low groups based on median values for expression levels or proportions of respective K-Ras isoforms (K-Ras4A and K-Ras4B) as shown in Additional file 1: Table S3. Each group was further divided into two subgroups according to the presence or absence of *KRAS* mutations, thus generating four groups for each K-Ras isoform. For intuitive notation of these groups, we designated high and low expression groups of the K-Ras4A isoform to KAexp^high^ and KAexp^low^, respectively, and denoted high and low proportion groups of the isoform to KAprop^high^ and KAprop^low^, respectively. In addition, we examined the mutation states (present or absent) of *EGFR* (G719A/C/S, exon 19 deletions, exon 20 insertions, S768I, T790 M, L858R, and L861Q), and *KRAS* (mutations at the 12th, 13th, and 61st codons) for all patients. We then denoted patient groups harboring mutant and wild type (wt) of *KRAS* and *EGFR* genes to *KRAS*^mut^ and *KRAS*^wt^; and *EGFR*^mut^ and *EGFR*^wt^, respectively. Patient group with or without *KRAS* amplification was designated to *KRAS*^amp(+)^ and *KRAS*^amp(−)^. Note that *KRAS*^amp(−)^ group includes patients with not only neutral but also decreased copy number of the gene.

### Statistical analysis

Overall survival (OS) and disease-free survival (DFS) rates were analyzed using the Kaplan-Meier method, and a log-rank test was used to compare the high and low groups. Cox regression analysis was performed with nine variables (age, gender, smoking history, K-Ras4A expression, K-Ras4A proportion, *KRAS* amplification, *KRAS* mutation, *EGFR* mutation, and pathological stage) to test for independent markers of OS. Age, gender, smoking history, and pathological stage were entered into the Cox proportional hazard model as class variables. The relationship between nominal variables was examined by Chi-square tests. The limit of significance for all analyses was defined with a *p* value of 0.05. Analyses were performed by using the statistical software R version 3.3.2.

## Results

### Expression levels and proportions of K-Ras isoforms

**Fig. 1:**
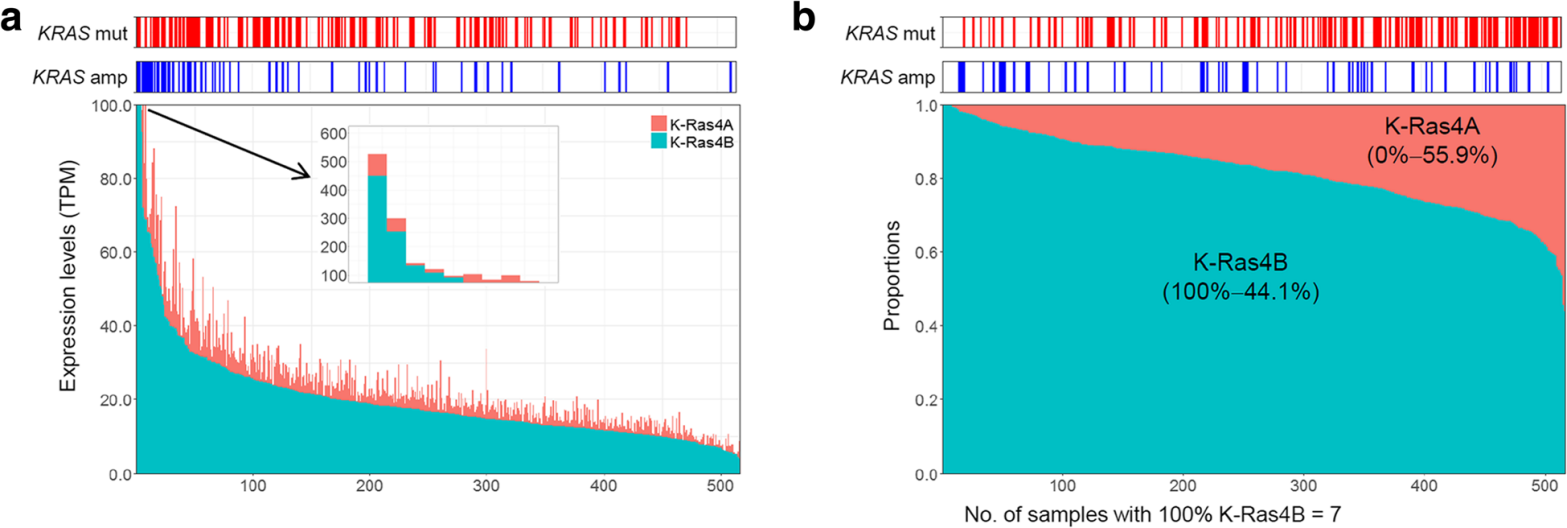
Isoform-level analyses of *KRAS* using the LUAD data set. Distribution of expression levels (**a**) and proportions (**b**) of K-Ras4A and K-Ras4B isoforms for LUAD patient samples, where *KRAS* mutations and amplification are represented to red and blue bars on the top of each panel

The distributions for expression levels (A) and proportions (B) of the two K-Ras isoforms for the LUAD cancer types are shown in Fig. 1, where the K-Ras4B isoform was observed as a major type as reported in a previous study [13]. Similar patterns were also observed for other cancer types (COAD, PAAD, and READ; Additional file 2: Figure S1). *KRAS* mutations and amplification are represented with red and blue bars, respectively, on the top of each panel in these figures. For *KRAS* mutations, we observed some trends with expression levels or proportions of a K-Ras isoform. For example, expression of the K-Ras4B isoform was increased according to increasing incidence rates of *KRAS* mutations in LUAD and READ (Fig. 1a and Additional file 2: Figure S1E, respectively). The proportions of the K-Ras4B isoform decreased according to increasing incidence rates of *KRAS* mutations in LUAD, but opposite was observed for the proportion of K-Ras4A (Fig. 1b). When we also checked for *KRAS* amplification, K-Ras4B expression seemed to have correlations with *KRAS* amplification in each cancer type unlikely proportions of both K-Ras isoforms (Fig. 1 and Additional file 2: Figure S1).

### Association between K-Ras isoforms and *KRAS* mutations

**Fig. 2:**
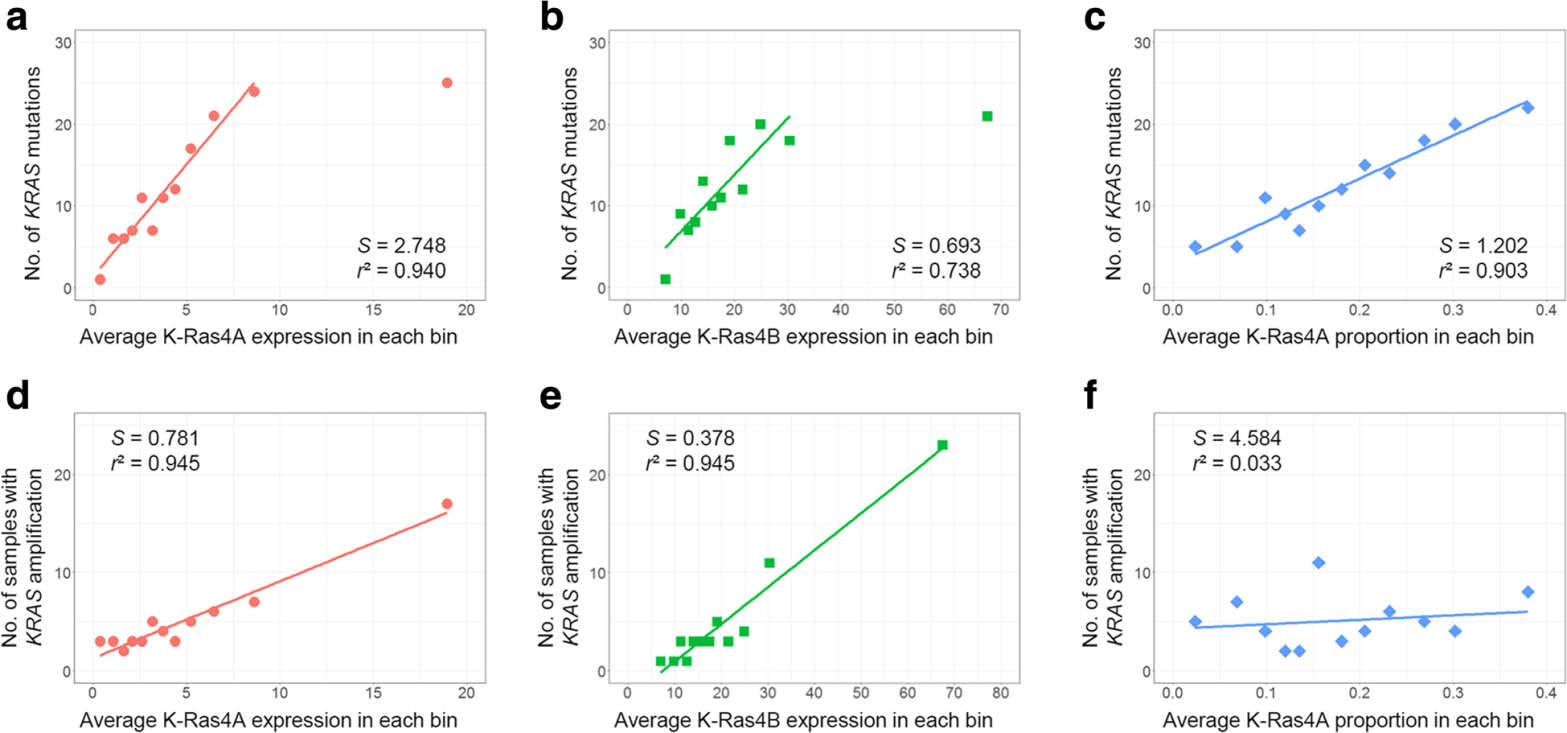
Linear regression analyses. The first (**a**, **b**, and **c**) and second rows (**d**, **e**, and **f**) indicate the results for *KRAS* mutations and amplification, respectively. The first (**a** and **d**), second (**b** and **e**), and third columns (**c** and **f**) represent the results for K-Ras4A expression, K-Ras4B expression, and K-Ras4A proportion, respectively. *S* and *r*^2^ indicate slope and R-squared, respectively

Next, we investigated how the degree of association between the K-Ras isoforms and *KRAS* mutations appeared in each cancer type via *r*^2^ and *S* values obtained from a linear regression analysis (Fig. 2a−c for LUAD and Additional file 2: Figure S2 for COAD, PAAD, and READ). When we examined for an association between K-Ras4A expression levels and the mutations, the LUAD and READ cancer types had *r*^2^ values (0.940 and 0.765, respectively) greater than the threshold of 0.6, of which only the *S* value for LUAD exceeded the threshold (2.748). For K-Ras4B expression levels versus the mutations, the *r*^2^ value for LUAD and READ (0.738 and 0.841, respectively) exceeded the threshold, but only *S* value for LUAD was greater than 0.6 (0.693). When we examined for the relationship between proportions of the two K-Ras isoforms and *KRAS* mutations, we selected the K-Ras4A instead of K-Ras4B isoform since K-Ras4A exhibited a positive association (positive *S* value), whereas the *r*^2^ values between the two isoforms were the same. While all cancer types had *S* values that exceeded the threshold, only the *r*^2^ value for LUAD was higher than the threshold (0.903). The above results revealed that the LUAD cancer type had a clear and strong association of not only expression levels, but also proportions of the K-Ras4A isoform with *KRAS* mutations.

### Association between K-Ras isoforms and *KRAS* amplification

We also examined association between isoforms and amplification of the gene via linear regression analysis in each cancer type (Fig. 2d−f for LUAD and Additional file 2: Figure S3 for COAD, PAAD, and READ). For K-Ras4A expression, LUAD showed strong association with *KRAS* amplification (*S* = 0.781, *r*^2^ = 0.945), but no or weak correlation was observed in other cancer types. For K-Ras4B expression, all cancer types exhibited more than moderate association (*r*^2^ > 0.6), of which the highest value was appeared in LUAD (*r*^2^ = 0.945). However, no correlation was found between K-Ras4A proportion and *KRAS* amplification (*r*^2^ < 0.2). Taken together, only *KRAS* amplification of LUAD cancer type showed strong association with expression of both K-Ras isoforms (*r*^2^ > 0.9), although *S* value was less than 0.6 for K-Ras4B expression.

### Correlation of K-Ras4A isoform with Clinicopathological parameters

We examined whether the K-Ras4A expression levels or proportions were associated with the clinicopathological parameters of LUAD patients as shown in Table 1. Both K-Ras4A expression levels and proportions were significantly associated with mutation states of *KRAS* (*p* = 4.82E-12 and *p* = 2.49E-07, respectively) and *EGFR* (*p* = 1.81E-3 and *p* = 0.0125, respectively) as well as the pathological stage (*p* = 0.0460 and *p* = 0.0327, respectively). Unlike *KRAS* mutation states, *KRAS* amplification was associated with K-Ras4A expression (*p* = 2.70E-3), but not for K-Ras4A proportion (*p* = 1.0). No association was observed with age (*p* = 1.0 and *p* = 0.616, respectively), gender (*p* = 1.0 and *p* = 0.791, respectively), and smoking history (*p* = 0.299 and *p* = 1.0, respectively). We confirmed that both K-Ras4A expression levels and proportions were correlated to the mutation states of well-known oncogenes (*KRAS* and *EGFR*) along with the pathological stages and only K-Ras4A expression levels were related to *KRAS* amplification. In addition, we found more significant *p* values in K-Ras4A expression than those in K-Ras4A proportion as following: 2.70E-3 versus 1.0 for *KRAS* amplification, 4.82E-12 versus 2.49E-7 for *KRAS* mutations, and 1.83E-3 versus 0.0125 for *EGFR* mutations, which seemed to suggest that K-Ras4A expression was stronger factor than K-Ras4A proportion.

**Table 1:** Baseline data of LUAD patients according to the expression and proportion of the K-Ras4A isoform

Clinical parameters	Values	All patients	K-Ras4A expression^a^			K-Ras4A proportion^b^		
		N (%)	High [N (%)]	Low [N (%)]	*p*-value^c^	High [N (%)]	Low [N (%)]	*p*-value
Age	≥65	276 (53.5)	137 (26.6)	139 (26.9)	1.0	141 (27.3)	135 (26.2)	0.616
	< 65	221 (42.8)	110 (21.3)	111 (21.5)		107 (20.7)	114 (22.1)	
	NA^d^	19 (3.7)	11 (2.1)	8 (1.6)		10 (1.9)	9 (1.9)	
Gender	Male	238 (46.1)	119 (23.1)	119 (23.1)	1.0	117 (22.7)	121 (23.4)	0.791
	Female	278 (53.9)	139 (26.9)	139 (26.9)		141 (27.3)	137 (26.6)	
Smoking history	Yes	427 (82.8)	219 (42.4)	208 (40.3)	0.299	215 (41.7)	212 (41.1)	1.0
	No	75 (14.5)	33 (6.4)	42 (8.1)		38 (7.4)	37 (7.2)	
	NA	14 (2.7)	6 (1.2)	8 (1.6)		5 (1.0)	9 (1.7)	
*KRAS* amplification^e^	Present	61 (11.8)	42 (8.1)	19 (3.7)	2.70E-3	30 (5.8)	31 (6.0)	1.0
	Absent	455 (88.2)	216 (41.9)	239 (46.3)		228 (44.2)	227 (44.0)	
*KRAS* mutations	Present	148 (28.7)	110 (21.3)	38 (7.4)	4.82E-12	101 (19.6)	47 (9.1)	2.49E-7
	Absent	368 (71.3)	148 (28.7)	220 (42.6)		157 (30.4)	211 (40.9)	
*EGFR* mutations	Present	45 (8.7)	12 (2.3)	33 (6.4)	1.81E-3	14 (2.7)	31 (6.0)	0.0125
	Absent	471 (91.3)	246 (47.7)	225 (43.6)		244 (47.3)	227 (44.0)	
Pathological stage	I	276 (53.5)	125 (24.2)	151 (29.3)	0.0460	128 (24.8)	148 (28.7)	0.0327
	II	122 (23.6)	66 (12.8)	56 (10.9)		58 (11.2)	64 (12.4)	
	III	84 (16.3)	52 (10.1)	32 (6.2)		54 (10.5)	30 (5.8)	
	IV	26 (5.0)	13 (2.5)	13 (2.5)		14 (2.7)	12 (2.3)	
	NA	8 (1.6)	2 (0.4)	6 (1.2)		4 (0.8)	4 (0.8)	

^a^Median value (3.41 TPM) of K-Ras4A expression levels was used to divide LUAD patients into two groups

^b^Median value (0.1659) of K-Ras4A proportions was used to divide LUAD patients into two groups

^c^Chi square tests were used to examine the relationship between the nominal variables

^d^NA, Not available

^e^The presence or absence of *KRAS* amplification were defined to 10% or more *KRAS* amplified or not

### OS according to *KRAS* mutation states

**Fig. 3:**
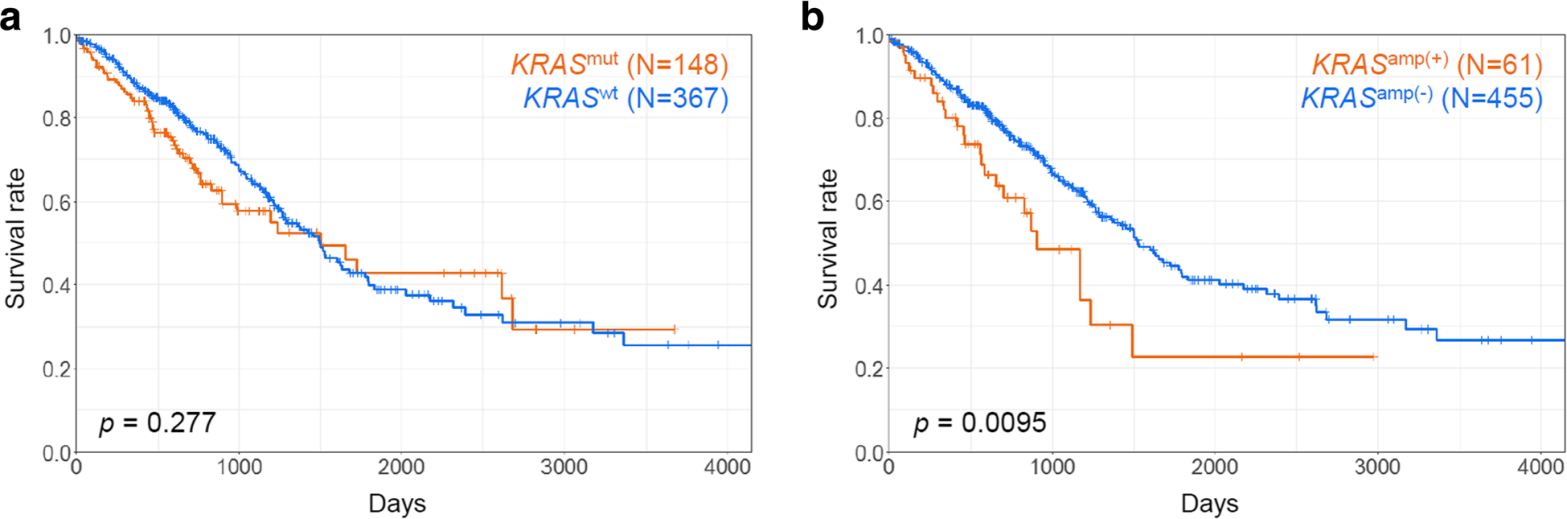
Overall survival of LUAD patients according to the states of *KRAS* mutations (**a**) and amplification (**b**). X- and y-axes represent survival time (days) and survival ratio, respectively

We examined the OS of LUAD patients according to the presence or absence of *KRAS* mutations, and confirmed that there were no significant differences in OS between the *KRAS*^mut^ and *KRAS*^wt^ groups (*p* = 0.227, Fig. 3a). We knew that patients with pathological stage I occupied more than 50% (276/516 [53.5%], Table 1). When we re-analyzed their OS with respect to pathological stages (I, II, or III/IV), no significant differences according to the mutation states were observed in patients with pathological stage I (*p* = 0.312, Additional file 2: Figure S4A). Accordingly, we assumed that the biased inclusion of the patients with pathological stage I might result in undistinguishable OS results according to the *KRAS* mutation states of all LUAD patients. By contrast, in cases of two patient groups with pathological stage II or III/IV, respectively, patients with *KRAS* mutations had relatively poor OS compared to patients with wild type *KRAS* although these results were not statistically significant (*p* = 0.145 for stage II, Additional file 2: Figure S4B; *p* = 0.153 for stage III/IV, Additional file 2: Figure S4C).

### OS according to *KRAS* amplification

We investigated OS according to the presence or absence of *KRAS* amplification for all LUAD patients. Significant poor outcome was observed in *KRAS*^amp(+)^ group compared to *KRAS*^amp(−)^ group (*p* = 9.50E-3, Fig. 3b). *Sasaki* et al. reported that *KRAS* mutation plus increased copy number was a predictor of poor clinical outcome in patients with NSCLC [11]. Accordingly, we examined their OS after dividing LUAD patient into three groups (*KRAS*^wt^/*KRAS*^amp(−)^, *KRAS*^mut^/*KRAS*^amp(+)^, and the remained patients). *KRAS*^mut^/*KRAS*^amp(+)^ group showed the worst prognosis among them, although the result was not statistically significant (*p* = 0.0872, Additional file 2: Figure S5).

### OS according to K-Ras4A isoform expression and proportion

**Fig. 4:**
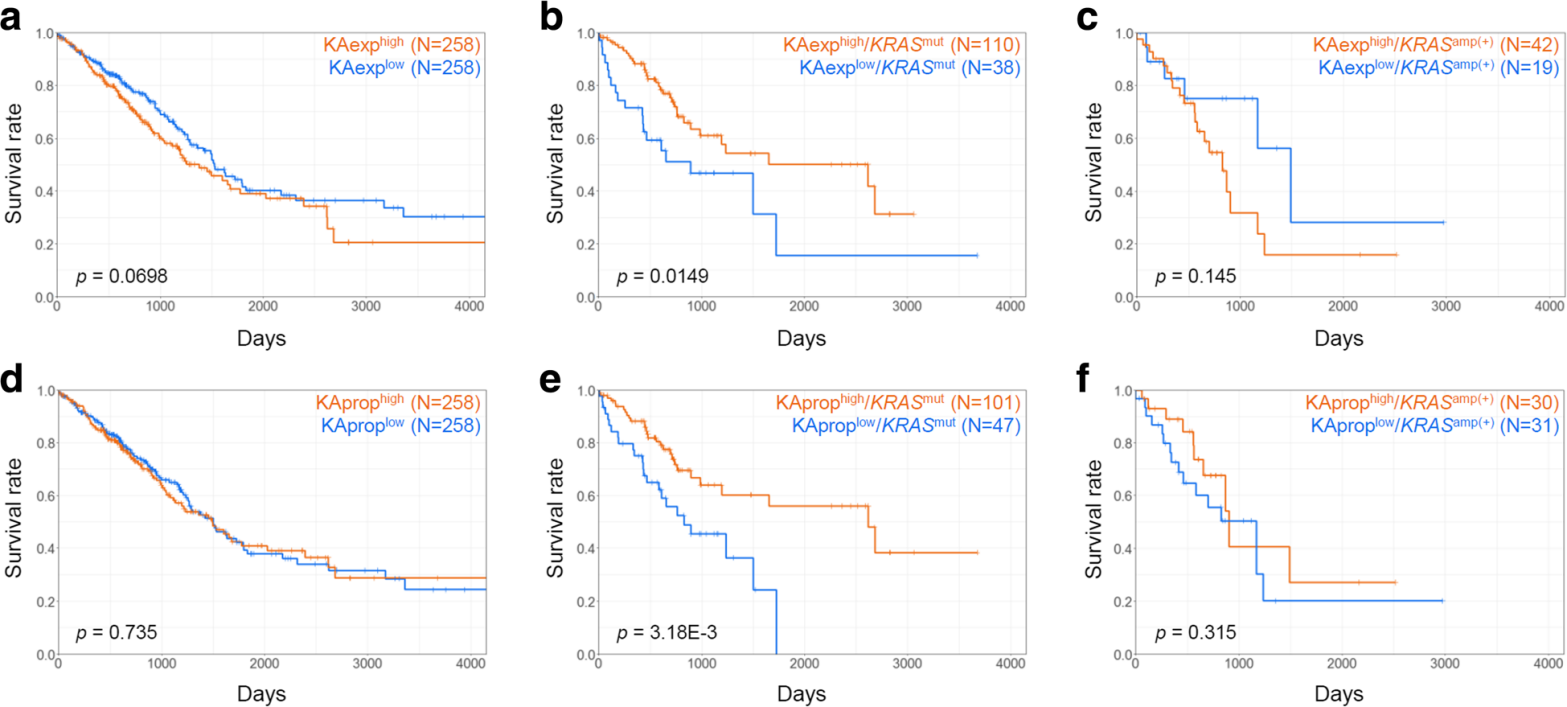
Overall survival of LUAD patients according to K-Ras4A expression and proportion. The first (**a**, **b**, and **c**) and second rows (**d**, **e**, and **f**) indicate the curves for the KAexp^high^ versus KAexp^low^ groups and KAprop^high^ versus KAprop^low^ groups, respectively. The first (**a** and **d**), second (**b** and **e**), and third columns (**c** and **f**) represent the curves for all patients, patients with *KRAS* mutations (*KRAS*^mut^ group), and patients with *KRAS* amplification (*KRAS*^amp(+)^ group), respectively. X- and y-axes represent survival time (days) and survival ratio, respectively

The Kaplan-Meier curves for the LUAD patients according to the expression levels or proportions of the K-Ras4A isoform are shown in Fig. 4. For all patients, there were no significant differences in OS between the two groups (*p* = 0.0698 for KAexp^high^ versus KAexp^low^, Fig. 4a; *p* = 0.735 for KAprop^high^ versus KAprop^low^, Fig. 4d). Since *KRAS* is a well-known oncogenic driver in lung cancer [3, 7], we further compared OS according to the mutational states of the gene in each group. In the *KRAS*^mut^ group, both KAexp^low^ and KAprop^low^ subgroups showed significantly worse OS than the KAexp^high^ and KAprop^high^ subgroups (*p* = 0.0149 and *p* = 3.18E-3, respectively; Fig. 4b, e). By checking the DFS, we confirmed that the OS results were convincing for the K-Ras4A expression (*p* = 3.03E-3, Additional file 2: Figure S6B) and proportion groups (*p* = 0.0237, Additional file 2: Figure S6E) for LUAD patients with *KRAS* mutations but not for all patients. Since *KRAS* amplification has been also identified as poor prognosis factor [11], OS of *KRAS*^amp(+)^ group was also examined and then compared with the results of *KRAS*^mut^ group. However, we did not observe significant OS results according to the high and low groups of K-Ras4A expression and proportion in the group (*p* = 0.145 and *p* = 0.315, respectively; Fig. 4c, f). This antagonistic result might be expected from the fact that only 27 LUAD patients simultaneously harbored both *KRAS* mutations and amplification. From these results, we questioned how were the LUAD patients with *KRAS* mutations divided into the two groups showing good and poor OS results according to K-Ras4A expression or proportion. Accordingly, we further examined the patient groups showing poor survival.

### Cox regression analysis

Using the Cox proportional hazards model, we investigated the hazard ratio (HR) of K-Ras4A expression and proportion in the LUAD cancer type. Table 2 shows the HRs and the significances of several covariates including K-Ras4A expression (A) or proportion (B) from uni- and multivariate analyses for all LUAD patients. The highest HR values were found in the subgroups for patients with pathological stage III/IV (HR = 2.486, 95% confidence interval [CI] = 1.796−3.443, and *p* = 4.14E-8 for expression groups; HR = 2.513, 95% CI = 1.815−3.480, and *p* = 2.90E-8 for proportion groups). For *EGFR* and *KRAS* mutations, HR values ranged from 1.246 to 1.460, which were not observed to be significant in both results. Under the same condition, we detected not only neutral HR values for K-Ras4A expression and proportion (0.900 and 1.049, respectively) but also a lack of their significances (0.537 and 0.770, respectively). These results were coincident with no significant OS and DFS differences between high and low groups for all LUAD patients (Fig. 4a, d and Additional file 2: Figure S6A, D). In contrast to mutation states of *EGFR* and *KRAS*, absence of *KRAS* amplification in all LUAD patients showed significant results when tested with K-Ras4A expression (HR = 0.623, 95% CI = 0.395−0.983, and *p* = 0.0421) and proportion (HR = 0.619, 95% CI = 0.393−0.977, and *p* = 0.0939), which were coincident with OS result as shown in Fig. 3b. Furthermore, results from the uni- and multivariate analyses for the patients with *KRAS* mutations (*KRAS*^mut^) are shown in Table 3. Pathological stage III/IV was also detected as a significant and independent factor with the highest HR value (HR = 3.268, 95% CI = 1.793−5.954, and *p* = 1.10E-4 for expression groups; HR = 3.404, 95% CI = 1.867−6.207, and *p* = 6.41E-5 for proportion groups). For *KRAS* amplification, non-significant *p* values (0.558 for expression groups and 0.903 for proportion groups) were observed unlikely the results from all LUAD patients, which were consisted with the results from survival analysis (Fig. 4c, f). In the same condition, both K-Ras4A expression and proportion had HR values greater than 2 (2.533 and 2.549, respectively) and appeared to be significant (2.72E-3 and 2.58E-3, respectively), thereby representing that they were equivalent independent markers for LUAD patients with *KRAS* mutations. Furthermore, these results were consistent with OS (Fig. 4b, e) and DFS results (Additional file 2: Figure S6B, E) for the corresponding patients. Finally, we examined the significance of the multivariate analysis for the intersect set of the two low groups showing poor survival by testing the following four groups: KAexp^high^/KAprop^high^ (*n* = 96); KAexp^high^/KAprop^low^ (*n* = 14); KAexp^low^/KAprop^high^ (*n* = 5); and KAexp^low^/KAprop^low^ (*n* = 33; Additional file 1: Table S4). As expected, the most significant group was identified to be KAexp^low^/KAprop^low^ (HR = 2.816, 95% CI = 1.453−5.459, and *p* = 2.17E-3), of which the statistical significance was similar to the above individual test results for K-Ras4A expression and proportion. Taken together, we suggest the possibility of both K-Ras4A expression and proportion to be utilized in survival predictions for LUAD patients with *KRAS* mutations. Note that one of the two factors should be used for the purpose, since moderate relationship (Pearson coefficient [*r*] = 0.505) was observed between K-Ras4A expression levels and proportions.

**Table 2:** Cox regression analysis for all LUAD patients

Covariates	A. K-Ras4A expression				Covariates	B. K-Ras4A proportion			
	Univariate analysis		Multivariate analysis			Univariate analysis		Multivariate analysis	
	HR^a^ (95% CI^b^)	*p*-value	HR (95% CI)	*p*-value		HR (95% CI)	*p*-value	HR (95% CI)	*p*-value
Age (≥65 years)	1.154 (0.857−1.553)	0.346	1.198 (0.879−1.632)	0.253	Age (≥65 years)	1.154 (0.857−1.553)	0.346	1.206 (0.885−1.643)	0.236
Gender (Male)	1.063 (0.797−1.417)	0.678	1.190 (0.871−1.625)	0.274	Gender (Male)	1.063 (0.797−1.417)	0.678	1.185 (0.867−1.619)	0.287
Smoking history	1.019 (0.887−1.170)	0.790	1.038 (0.895−1.202)	0.625	Smoking history	1.019 (0.887−1.170)	0.790	1.039 (0.896−1.204)	0.615
K-Ras4A expression (low)	0.766 (0.573−1.023)	0.0705	0.900 (0.644−1.257)	0.537	K-Ras4A proportion (low)	0.952 (0.714−1.268)	0.735	1.049 (0.762−1.444)	0.770
*KRAS* amplification (Not amplified)	0.580 (0.382−0.880)	0.0104	0.623 (0.395−0.983)	0.0421	*KRAS* amplification (Not amplified)	0.580 (0.382−0.880)	0.0104	0.619 (0.393−0.977)	0.0393
*KRAS* mutations (Present)	1.194 (0.867−1.643)	0.277	1.246 (0.856−1.813)	0.252	*KRAS* mutations (Present)	1.194 (0.867−1.643)	0.277	1.319 (0.915−1.901)	0.138
*EGFR* mutations (Present)	1.322 (0.831−2.104)	0.239	1.460 (0.869−2.454)	0.153	*EGFR* mutations (Present)	1.322 (0.831−2.104)	0.239	1.436 (0.855−2.412)	0.172
Pathological stage (III/IV)	2.635 (1.939−3.581)	5.94E-10	2.486 (1.796−3.443)	4.14E-8	Pathological stage (III/IV)	2.635 (1.939−3.581)	5.94E-10	2.513 (1.815−3.480)	2.90E-8

^a^HR, hazard ratio

^b^CI, confidence interval

**Table 3:** Cox regression analysis for LUAD patients harboring *KRAS* mutations

Covariates	A. K-Ras4A expression				Covariates	B. K-Ras4A proportion			
	Univariate analysis		Multivariate analysis			Univariate analysis		Multivariate analysis	
	HR^a^ (95% CI^b^)	*p*-value	HR (95% CI)	*p*-value		HR (95% CI)	*p*-value	HR (95% CI)	*p*-value
Age (≥65 years)	1.326 (0.761−2.312)	0.320	1.351 (0.735−2.480)	0.333	Age (≥65 years)	1.326 (0.761−2.312)	0.320	1.443 (0.778−2.678)	0.245
Gender (Male)	1.536 (0.891−2.650)	0.123	1.734 (0.954−3.152)	0.0711	Gender (Male)	1.536 (0.891−2.650)	0.123	1.690 (0.911−3.061)	0.0971
Smoking history	1.076 (0.804−1.440)	0.622	1.168 (0.851−1.602)	0.337	Smoking history	1.076 (0.804−1.440)	0.622	1.191 (0.866−1.638)	0.282
*KRAS* amplification (Not amplified)	0.820 (0.410−1.643)	0.576	0.802 (0.383−1.679)	0.558	*KRAS* amplification (Not amplified)	0.820 (0.410−1.643)	0.576	0.954 (0.448−2.031)	0.903
K-Ras4A expression (low)	1.986 (1.131−3.490)	0.017	2.533 (1.380−4.651)	2.72E-3	K-Ras4A proportion (low)	2.258 (1.295−3.938)	4.10E-3	2.549 (1.387−4.684)	2.58E-3
Pathological stage (III/IV)	3.088 (1.747−5.459)	1.05E-4	3.268 (1.793−5.954)	1.10E-4	Pathological stage (III/IV)	3.088 (1.747−5.459)	1.05E-4	3.404 (1.867−6.207)	6.41E-5

^a^HR, hazard ratio

^b^CI, confidence interval

## Discussion

The advents of RNA-seq technology and relevant bioinformatics tools have enabled us to perform isoform-level expression analyses via reliable identification of the isoforms. The approach has been used for various kinds of cancer research including the detection of tumor-specific isoforms [24], thereby enabling the identification of potential biomarkers for clinical purposes including diagnosis [24, 25]. In this study, we presented another utility of isoform-level analysis that enabled a possible predictive role of abundance measures for the K-Ras4A isoform on the survival of LUAD patients harboring *KRAS* mutations. In light of the statistical significance, we anticipate that isoform-level abundance of genes may confer a new factor for a deeper consideration of patient prognosis and stratification in cancer.

A recent study reported that *KRAS* was one of the genes showing significantly positive variant allele frequencies in RNA compared to DNA [12]. Accordingly, it was needed to check whether each K-Ras isoform was expressed from mutant or wild type alleles in the condition of allelic imbalance of *KRAS* transcription. Current paired-end sequencing technology makes it possible to achieve the purpose by examining read pairs. But direct investigation in the cancer type was impossible due to the absence of raw RNA-seq data. Instead, we examined the corresponding read pairs in other lung adenocarcinoma data set generated by paired-end sequencing (GSE81089 [26]). Because relatively long fragment sizes are required for *KRAS* mutations at 12th or 13th codons than that at 61st codon, we could observe one or two read pairs in the former cases, while several read pairs were found in the latter case (Additional file 2: Figure S7). Since this examination is depend on the degree of fragmentation before sequencing, excessive fragmentation of template RNA molecules during library preparation will be complicated to confirm both *KRAS* mutation status and isoform origin of the read pairs. Indeed, we could not find them due to the lack of fragments satisfying the minimal size in another lung adenocarcinoma data set (GSE40419 [27]; data not shown).

While our analysis has been conducted based on a robust measurement of gene expression in a sufficiently large-scale cohort, there are some intrinsic limitations that may affect the interpretation and the reproducibility of the study. Generally, the causal relation between the molecular factor (here, K-Ras expression) and the survival (OS) can be hardly drawn in cohort-based studies, which can weaken the functional association. Moreover, the use and the definition of OS can be different by the study design. For example, there are four distinct uses of OS as a metric: i) survival time as a patient outcome, ii) patient survival as a therapeutic objective, iii) OS as a trial endpoint, and iv) survival as a public health measure [28]. The OS of patients who participated in the TCGA project [18] might have been used as a therapeutic metric by clinicians to track their survival. Accordingly, the OS used in this study could be affected by several factors including pathological states and individual responses for chemo- and/or targeted therapies. Further studies are also needed to confirm whether our findings are also observed in other lung cancer data sets before being applied in clinical practice. Finally, the lack of proper independent cohort for validation is a remaining hurdle. As known, TCGA is the currently largest cohort that provides multi-omics data with well-defined clinical information. Based on an extensive search, we found one and the only available independent cohort with raw RNA-seq data and survival information (GSE81089 [26]). However, application of our analysis on the cohort was unsuccessful, due to the smaller cohort size (*n* = 108, compared to 516 in TCGA) and low sample purity criteria (> 10%, compared to > 50% in TCGA). We would like to note that the purity of sample is extremely important to measuring gene expression of cancer cells, because inclusion of normal cells (e.g., stromal cells) can perplex the inference of cancer specific mRNA abundance. We anticipate that the completion and the public distribution of currently ongoing large-scale genomic projects such as International Cancer Genome Consortium [29] and Genomics Evidence Neoplasia Information Exchange [30] will lead to a proper evaluation of the association between isoform expression and cancer prognosis.

## Conclusions

By performing an isoform-level analysis, we found two abundance measures (expression and proportion) for the K-Ras4A isoform that were associated with survival rate of patients with both LUAD and *KRAS* mutations. We showed their possibility in predicting lung cancer survival rates by identifying their roles as independent prognostic markers through multivariate analysis. Furthermore, we demonstrated that isoform-level analysis was a very useful approach in identifying hidden factors that can be utilized in the clinic.

## Additional files

Additional file 1: **Table S1.** Somatic mutationand RNA-seq data files for LUAD. **Table S2.** Somatic mutation and RNA-seq data files for COAD, PAAD, and READ. **Table S3.** Median values of expression levels orproportions of K-Rasisoforms used for patient grouping. **Table S4.** Combined result of Cox regression analysis according to the states of K-Ras4A expression and proportionfor LUAD patients with KRAS mutations. (PDF 470 kb)

Additional file 2: **Figure S1.** Expression and proportion of K-Ras4A and K-Ras4B isoforms in COAD, PAAD, and READ. **Figure S2.** Linear regression analysis of KRAS mutations versus K-Ras4A expression, K-Ras4B expression, or K-Ras4A proportion in COAD, PAAD, and READ. **Figure S3.** Linear regression analysis of *KRAS* amplification versus K-Ras4A expression, K-Ras4B expression, or K-Ras4A proportion in COAD, PAAD, and READ cancer types. **Figure S4.** Overall survival of LUAD patients according to mutation states of *KRAS* mutations and pathological stages. **Figure S5.** Overall survival of LUAD patients according to the states of *KRAS* mutations and amplification. **Figure S6.** Disease-free survival of LUAD patients according to K-Ras4A expression and proportion. **Figure S7.** Integrative Genomics Viewer screen shots of aligned reads for three RNA-seq data sets of patients with *KRAS* mutations (SRX1741889, G12S (A); SRX174187, G13D (B); and SRX1741936, Q61H (C)). (PDF 638 kb)

## Abbreviations

CI: Confidence interval
COAD: Colon adenocarcinoma
DFS: Disease-free survival
EGFR: Epidermal growth factor receptor
HR: Hazard ratio
KAexp^high^: Patients with high K-Ras4A expression
KAexp^low^: Patients with low K-Ras4A expression
KAprop^high^: Patients with high K-Ras4A proportion
KAprop^low^: Patients with low K-Ras4A proportion
*KRAS*: Kirsten rat sarcoma viral oncogene homolog
*KRAS*^amp(−)^: Patients without *KRAS* amplification
*KRAS*^amp(+)^: Patients with *KRAS* amplification
*KRAS*^mut^: Patients with *KRAS* mutations
*KRAS*^wt^: Patients with wild type *KRAS*
LUAD: Lung adenocarcinoma
OS: Overall survival
PAAD: Pancreatic adenocarcinoma
READ: Rectal adenocarcinoma
RNA-seq: Whole transcriptome sequencing
TCGA: The Cancer Genome Atlas
TPM: Transcript per million
