# Supplementary material for: Isoform specific gene expression analysis of KRAS in the prognosis of lung adenocarcinoma patients
Source: BMC Bioinformatics. 2018 Feb 19;19(Suppl 1):40. doi: 10.1186/s12859-018-2011-y (PMC6389035; doi:10.1186/s12859-018-2011-y)
Supplement: Supplementary file 1 — Table S1. Somatic mutationand RNA-seq data files for LUAD. Table S2. Somatic mutation and RNA-seq data files for COAD, PAAD, and READ. Table S3. Median values of expression levels orproportions of K-Rasisoforms used for patient grouping. Table S4. Combined result of Cox regression analysis according to the states of K-Ras4A expression and proportionfor LUAD patients with KRAS mutations. (PDF 470 kb) [file 12859_2018_2011_MOESM1_ESM.pdf]

Research Article

Isoform specific gene expression analysis of *KRAS* in the prognosis of lung adenocarcinoma patients

In Seok Yang[1] and Sangwoo Kim[1,*]

[1]Severance Biomedical Science Institute, Yonsei University College of Medicine, 50-1 Yonsei-ro, Seoul 03722, Korea

*Corresponding author:

Sangwoo Kim, Ph.D.

Tel: +82-2-2228-0913

Email: swkim@yuhs.ac

Running title: Isoform specific expression of *KRAS*

Supplementary Information

Table S1. Somatic mutation and RNA-seq data files downloaded for the LUAD cancer type from the TCGA Data Portal (https://tcga-data.nci.nih.gov/docs/publications/tcga/), which can be currently found in Genomic Data Commons Legacy Archive (https://portal.gdc.cancer.gov/legacy-archive/search/f) maintained by the National Cancer Institute.

Cancer types	Data types	Levels	Files
LUAD	Somatic mutation	2.1.5.0	PR_TCGA_LUAD_PAIR_Capture_All_Pairs_QCPASS_v4.aggregated.capture.tcga.uuid.automated.somatic.maf
	RNA-seq	3.1.14.0	unc.edu_LUAD.IlluminaHiSeq_RNASeqV2.Level_3.1.14.0
		3.1.0.0	unc.edu_LUAD.IlluminaHiSeq_TotalRNASeqV2.Level_3.1.0.0

LUAD, lung adenocarcinoma

Table S2. Somatic mutation and RNA-seq data files downloaded for COAD, PAAD, and READ cancer types from the TCGA Data Portal (https://tcga-data.nci.nih.gov/docs/publications/tcga/), which are currently found in the Genomic Data Commons Legacy Archive (https://portal.gdc.cancer.gov/legacy-archive/search/f) maintained by National Cancer Institute.

Cancer types	Data types	Levels	Files
COAD	Somatic mutation	3.1.1.0	unc.edu_COAD.IlluminaGA_RNASeqV2.Level_3.1.1.0
		3.1.12.0	unc.edu_COAD.IlluminaHiSeq_RNASeqV2.Level_3.1.12.0
		3.1.0.0	unc.edu_COAD.IlluminaHiSeq_TotalRNASeqV2.Level_3.1.0.0
	RNA-seq	2.1.5.0	hgsc.bcm.edu_COAD.IlluminaGA_DNASeq.1.somatic.maf
PAAD	Somatic mutation	2.1.10.0	PAAD_pairs.aggregated.capture.tcga.uuid.automated.somatic.maf
	RNA-seq	3.1.8.0	unc.edu_PAAD.IlluminaHiSeq_RNASeqV2.Level_3.1.8.0
READ	Somatic mutation	2.1.6.0	hgsc.bcm.edu_READ.IlluminaGA_DNASeq.1.somatic.maf
	RNA-seq	3.1.0.0	unc.edu_READ.IlluminaGA_RNASeqV2.Level_3.1.0.0
		3.1.9.0	unc.edu_READ.IlluminaHiSeq_RNASeqV2.Level_3.1.9.0

COAD, colon adenocarcinoma; PAAD, pancreatic adenocarcinoma; READ, rectal adenocarcinoma.

Table S3. Median values of expression levels or proportions of K-Ras isoforms, which were used to divide patients of each cancer type into two groups

Cancer types	K-Ras4A isoform		K-Ras4B isoform	
	Expression levels (TPM)	Proportions	Expression levels (TPM)	Proportions
COAD	3.4652805	0.2197325	12.105005	0.7802675
LUAD	3.408776	0.1659475	16.557391	0.8340525
PAAD	3.558625	0.207049	13.170599	0.792951
READ	3.071302	0.210092	11.38283	0.789908

COAD, colon adenocarcinoma; LUAD, lung adenocarcinoma; PAAD, pancreatic adenocarcinoma; READ, rectal adenocarcinoma.

Table S4. Combined result of Cox regression analysis according to the states of K-Ras4A expression and proportion for LUAD patients with *KRAS* mutations

Covariates	Combined result of K-Ras4A expression and proportion			
	Univariate analysis		Multivariate analysis	
	HR (95% CI)	*p*-value	HR (95% CI)	*p*-value
Age (≥65)	1.326 (0.761–2.312)	0.320	1.456 (0.786–2.697)	0.232
Gender (Male)	1.536 (0.891–2.650)	0.123	1.733 (0.949–3.164)	0.0737
Smoking history	1.076 (0.804–1.440)	0.622	1.181 (0.858–1.627)	0.308
K-Ras4A expression and proportion (High/Low)	2.009 (0.866–4.661)	0.104	2.584 (1.015–6.578)	0.0464
K-Ras4A expression and proportion (Low/High)	1.318 (0.307–5.663)	0.710	4.905 (1.110–21.684)	0.0360
K-Ras4A expression and proportion (Low/Low)	2.427 (1.309–4.501)	4.88E-3	2.816 (1.453–5.459)	2.17E-3
Pathological stage (III/IV)	3.088 (1.747–5.459)	1.05E-04	3.483 (1.903–6.376)	5.23E-5

HR, hazard ratio; CI, confidence interval.
