# Supplementary material for: Isoform specific gene expression analysis of KRAS in the prognosis of lung adenocarcinoma patients
Source: BMC Bioinformatics. 2018 Feb 19;19(Suppl 1):40. doi: 10.1186/s12859-018-2011-y (PMC6389035; doi:10.1186/s12859-018-2011-y)
Supplement: Supplementary file 2 — Figure S1. Expression and proportion of K-Ras4A and K-Ras4B isoforms in COAD, PAAD, and READ. Figure S2. Linear regression analysis of KRAS mutations versus K-Ras4A expression, K-Ras4B expression, or K-Ras4A proportion in COAD, PAAD, and READ. Figure S3. Linear regression analysis of KRAS amplification versus K-Ras4A expression, K-Ras4B expression, or K-Ras4A proportion in COAD, PAAD, and READ cancer types. Figure S4. Overall survival of LUAD patients according to mutation states of KRAS mutations and pathological stages. Figure S5. Overall survival of LUAD patients according to the states of KRAS mutations and amplification. Figure S6. Disease-free survival of LUAD patients according to K-Ras4A expression and proportion. Figure S7. Integrative Genomics Viewer screen shots of aligned reads for three RNA-seq data sets of patients with KRAS mutations (SRX1741889, G12S (A); SRX174187, G13D (B); and SRX1741936, Q61H (C)). (PDF 638 kb) [file 12859_2018_2011_MOESM2_ESM.pdf]

LEGENDS OF SUPPLEMENTAL FIGURES

Figure S1. Expression and proportion of K-Ras4A and K-Ras4B isoforms in COAD, PAAD, and READ cancer types. The first (A, C, and E) and second (B, D, and F) columns of panels show distributions of expression and proportion of the two K-Ras isoforms, respectively. The first (A and B), second (C and D), and third (E and F) rows of panels depict results for COAD, PAAD, and READ cancer types, respectively. *KRAS* mutations and amplification are represented to red and blue bars on the top of each panels, respectively.

Figure S2. Linear regression analysis of *KRAS* mutations versus K-Ras4A expression, K-Ras4B expression, or K-Ras4A proportion in COAD, PAAD, and READ cancer types. The first (A, D, and G), second (B, E, and H), and third (C, F, and I) columns of panels show results for K-Ras4A expression, K-Ras4B expression, and K-Ras4A proportion, respectively. The first (A, B, and C), second (D, E, and F), and third rows (G, H, and I) rows of panels depict results for COAD, PAAD, and READ cancer types, respectively. X-axes in the first and second columns of panels represent the average expression levels of K-Ras4A and K-Ras4B, respectively, in each bin. X-axes in the third column of panels represent the average proportion of K-Ras4A. Y-axes represent the sum of *KRAS* mutations in each bin. S and r^2 indicate slope and R-squared, respectively.

Figure S3. Linear regression analysis of *KRAS* amplification versus K-Ras4A expression, K-Ras4B expression, or K-Ras4A proportion in COAD, PAAD, and READ cancer types. The first (A, D, and G), second (B, E, and H), and third (C, F, and I) columns of panels show results for K-Ras4A expression, K-Ras4B expression, and K-Ras4A proportion, respectively. The first (A, B, and C), second (D, E, and F), and third rows (G, H, and I) rows of panels depict results for COAD, PAAD, and READ cancer types, respectively. X-axes in the first and second columns of panels represent the average expression levels of K-Ras4A and K-Ras4B, respectively, in each bin. X-axes in the third column of panels represent the average proportion of K-Ras4A. Y-axes represent the number of samples with *KRAS* amplification in each bin. S and r^2 indicate slope and R-squared, respectively.

Figure S4. Overall survival of LUAD patients according to mutation states of KRAS mutations and pathological stages. Patients with stage I (A), patients with stage II (B), and patients with stage III/IV (C). X- and y-axes represent survival time (days) and survival ratio, respectively.

LEGENDS OF SUPPLEMENTAL FIGURES (continued)

Figure S5. Overall survival of LUAD patients according to the states of *KRAS* mutations and amplification. "MA" and "N" represent *KRAS*^{mut}/*KRAS*$^{amp(+)}$ and *KRAS*^{wt}/*KRAS*$^{amp(-)}$ patient groups, respectively. "M or A" indicates *KRAS*^{mut}/*KRAS*$^{amp(-)}$ or *KRAS*^{wt}/*KRAS*$^{amp(+)}$ patient groups.

Figure S6. Disease-free survival of LUAD patients according to K-Ras4A expression and proportion. The first (A, B, and C) and second rows (D, E, and F) indicate the curves for the KAexp^{high} versus KAexp^{low} groups and KAprop^{high} versus KAprop^{low} groups, respectively. The first (A and D), second (B and E), and third columns (C and F) represent the curves for all patients, patients with *KRAS* mutations (*KRAS*^{mut} group), and patients with *KRAS* amplification (*KRAS*$^{amp(+)}$ group), respectively. X- and y-axes represent survival time (days) and survival ratio, respectively.

Figure S7. Integrative Genomics Viewer screen shots of aligned reads for three RNA-seq data sets of patients with *KRAS* mutations (SRX1741889, G12S (A); SRX174187, G13D (B); and SRX1741936, Q61H (C)). *KRAS* mutation status and isoform origin were simultaneously identified in one or two read pairs in the cases of G12S and G13D mutations, but several read pairs were found in the case of Q61H mutation.

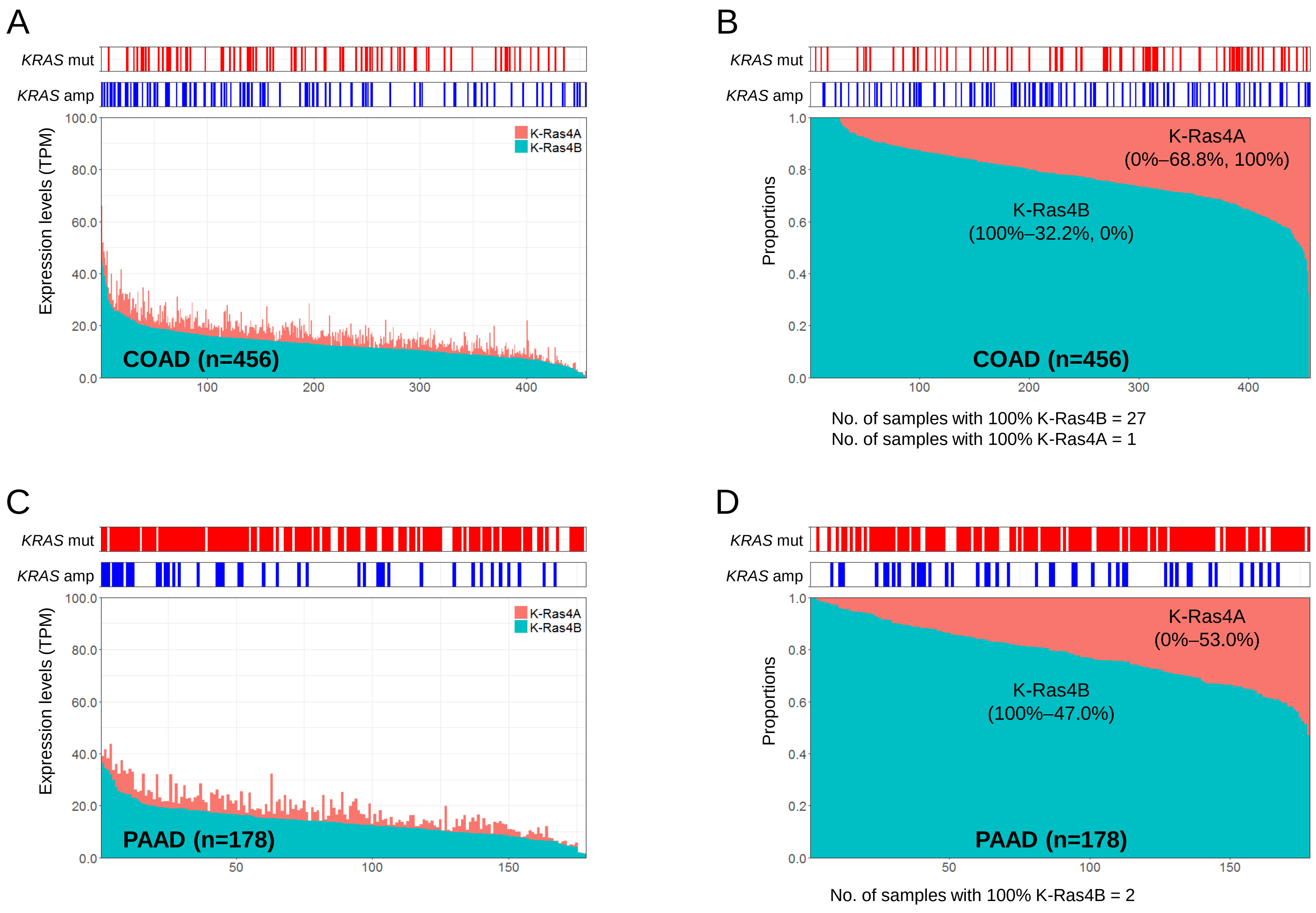
A
KRAS mut
KRAS amp
Expression levels (TPM)
K-Ras4A
K-Ras4B
COAD (n=456)
B
KRAS mut
KRAS amp
Proportions
K-Ras4A
(0%–68.8%, 100%)
K-Ras4B
(100%–32.2%, 0%)
COAD (n=456)
No. of samples with 100% K-Ras4B = 27
No. of samples with 100% K-Ras4A = 1
C
KRAS mut
KRAS amp
Expression levels (TPM)
K-Ras4A
K-Ras4B
PAAD (n=178)
D
KRAS mut
KRAS amp
Proportions
K-Ras4A
(0%–53.0%)
K-Ras4B
(100%–47.0%)
PAAD (n=178)
No. of samples with 100% K-Ras4B = 2

Figure S1

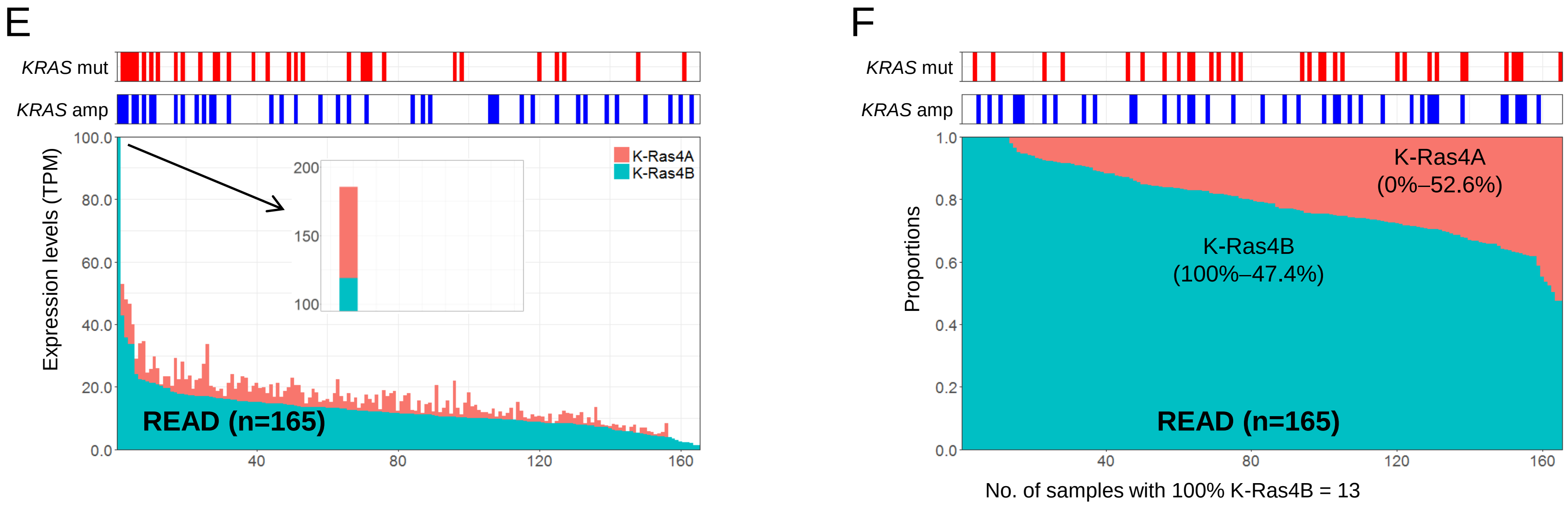

Figure S1 (continued)

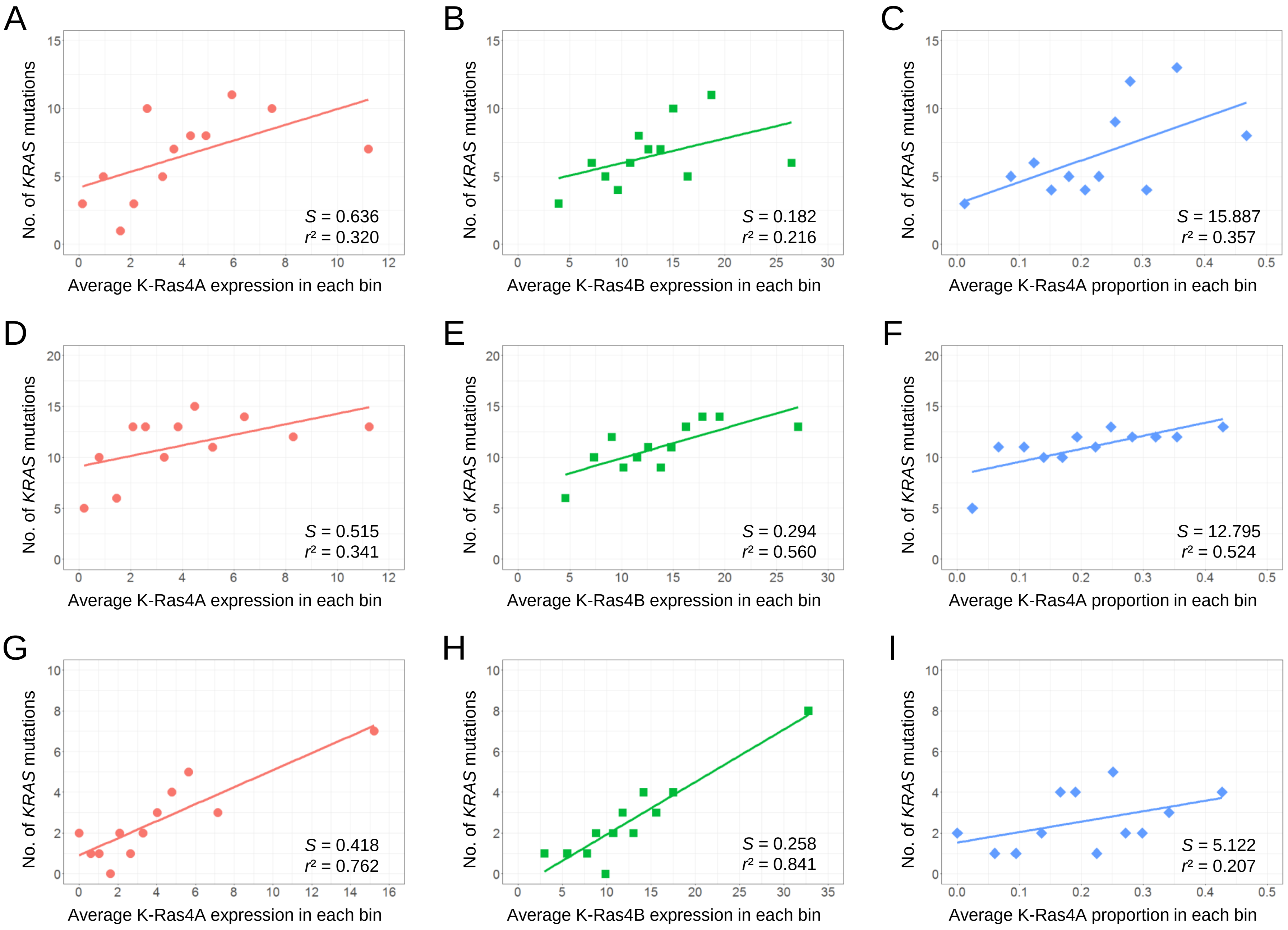

A
B
C
D
E
F
G
H
I
No. of KRAS mutations
Average K-Ras4A expression in each bin
Average K-Ras4B expression in each bin
Average K-Ras4A proportion in each bin
S = 0.636
r² = 0.320
S = 0.182
r² = 0.216
S = 15.887
r² = 0.357
S = 0.515
r² = 0.341
S = 0.294
r² = 0.560
S = 12.795
r² = 0.524
S = 0.418
r² = 0.762
S = 0.258
r² = 0.841
S = 5.122
r² = 0.207

Figure S2

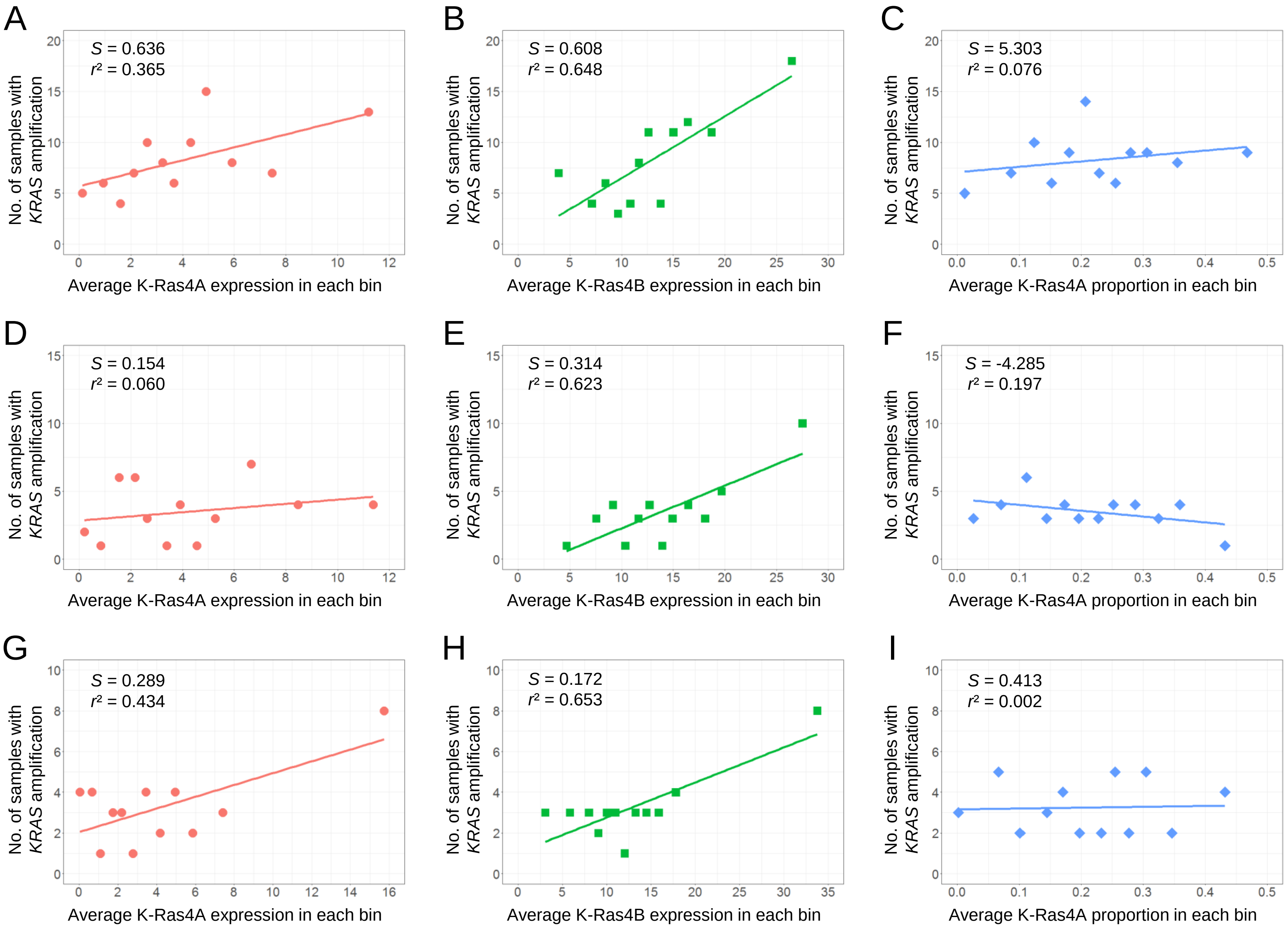
A
S = 0.636
r² = 0.365
B
S = 0.608
r² = 0.648
C
S = 5.303
r² = 0.076
D
S = 0.154
r² = 0.060
E
S = 0.314
r² = 0.623
F
S = -4.285
r² = 0.197
G
S = 0.289
r² = 0.434
H
S = 0.172
r² = 0.653
I
S = 0.413
r² = 0.002
No. of samples with KRAS amplification
Average K-Ras4A expression in each bin
Average K-Ras4B expression in each bin
Average K-Ras4A proportion in each bin

Figure S3

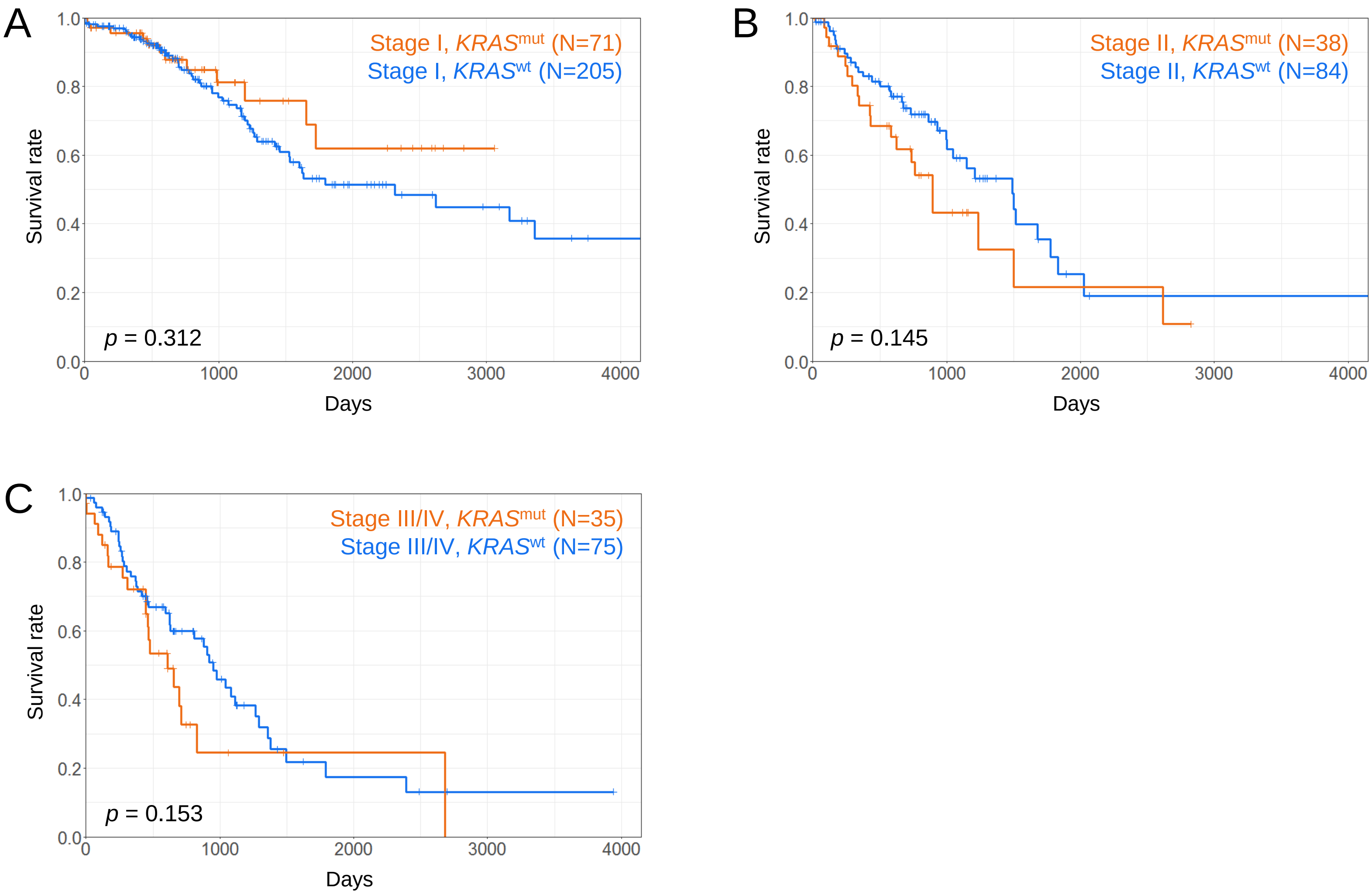
A
Stage I, KRASmut (N=71)
Stage I, KRASwt (N=205)
p = 0.312
Survival rate
Days
B
Stage II, KRASmut (N=38)
Stage II, KRASwt (N=84)
p = 0.145
Survival rate
Days
C
Stage III/IV, KRASmut (N=35)
Stage III/IV, KRASwt (N=75)
p = 0.153
Survival rate
Days

Figure S4

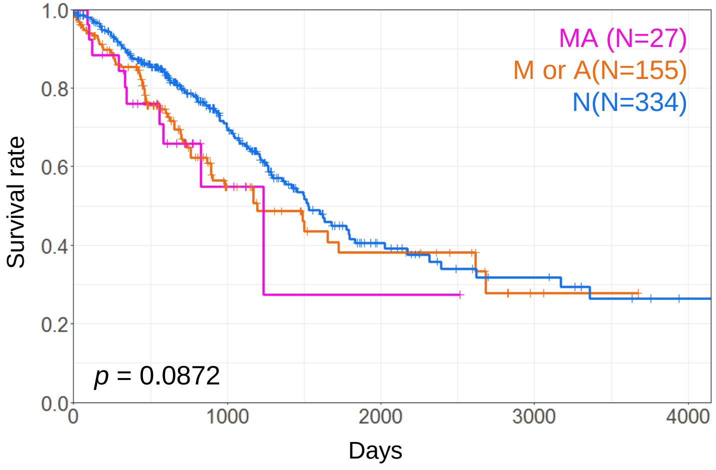
MA (N=27)
M or A(N=155)
N(N=334)
Survival rate
1.0
0.8
0.6
0.4
0.2
0.0
p = 0.0872
0
1000
2000
3000
4000
Days

Figure S5

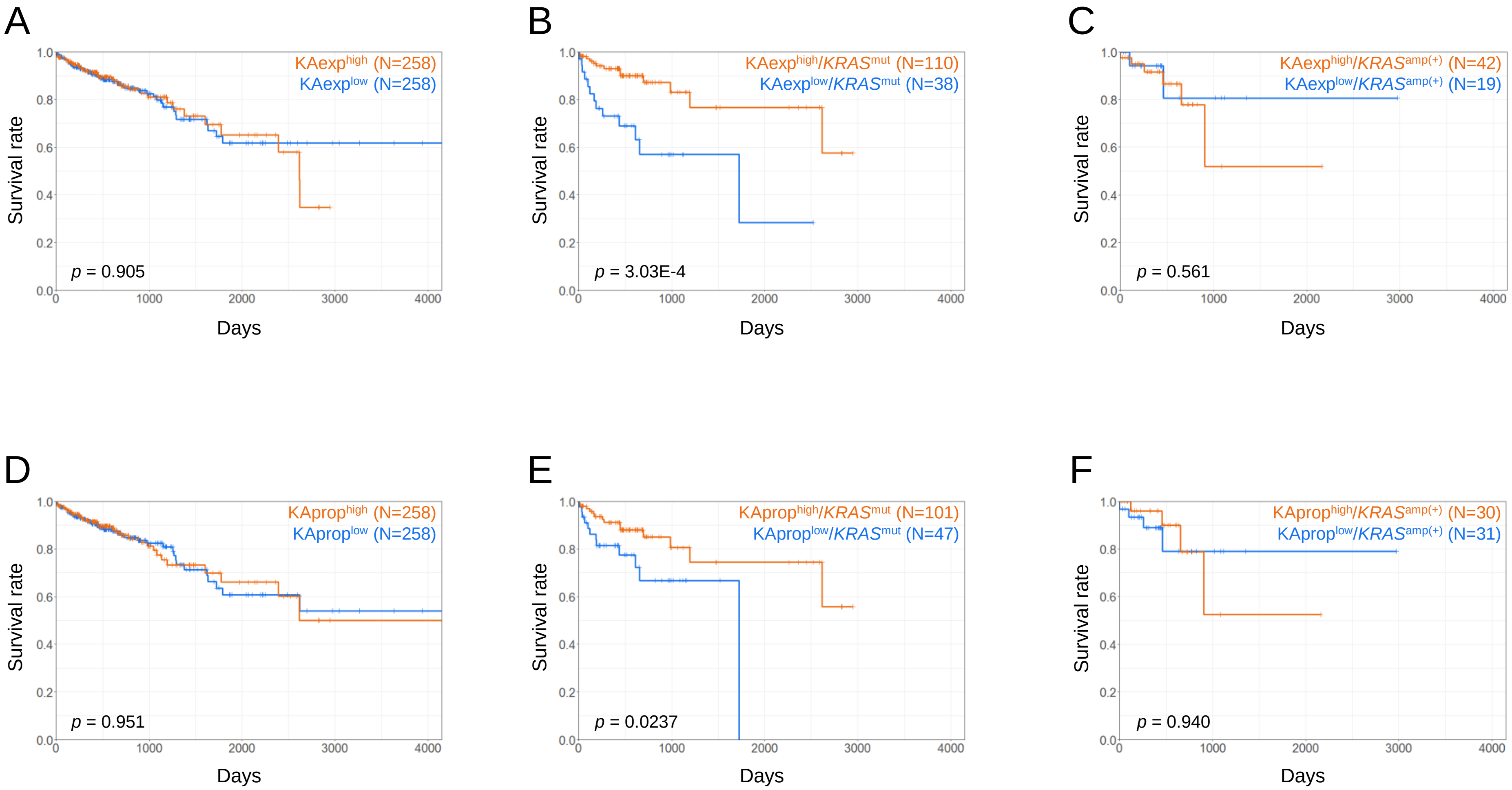
A
KAexp^high (N=258)
KAexp^low (N=258)
p = 0.905
B
KAexp^high/KRAS^mut (N=110)
KAexp^low/KRAS^mut (N=38)
p = 3.03E-4
C
KAexp^high/KRAS^amp(+) (N=42)
KAexp^low/KRAS^amp(+) (N=19)
p = 0.561
D
KAprop^high (N=258)
KAprop^low (N=258)
p = 0.951
E
KAprop^high/KRAS^mut (N=101)
KAprop^low/KRAS^mut (N=47)
p = 0.0237
F
KAprop^high/KRAS^amp(+) (N=30)
KAprop^low/KRAS^amp(+) (N=31)
p = 0.940
Survival rate
Days
0.0
0.2
0.4
0.6
0.8
1.0
0
1000
2000
3000
4000

Figure S6

A SRX1741889 (G12S)

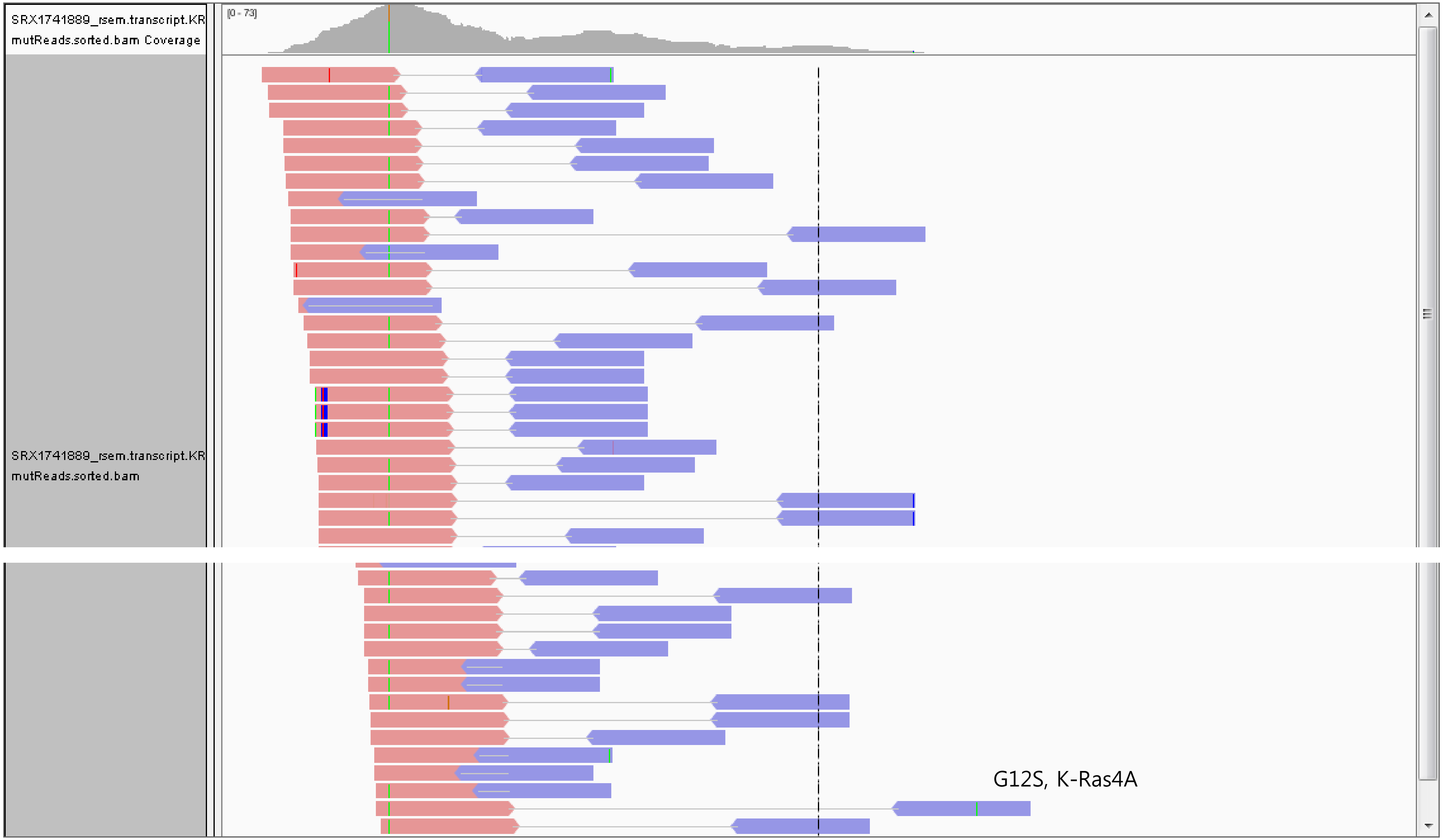

Only one read pair was confirmed for both *KRAS* mutation status and isoform origin. The remained pairs were unknown for whether they were originated from K-Ras4A or K-Ras4B isoforms.

Figure S7

B SRX1741877 (G13D)

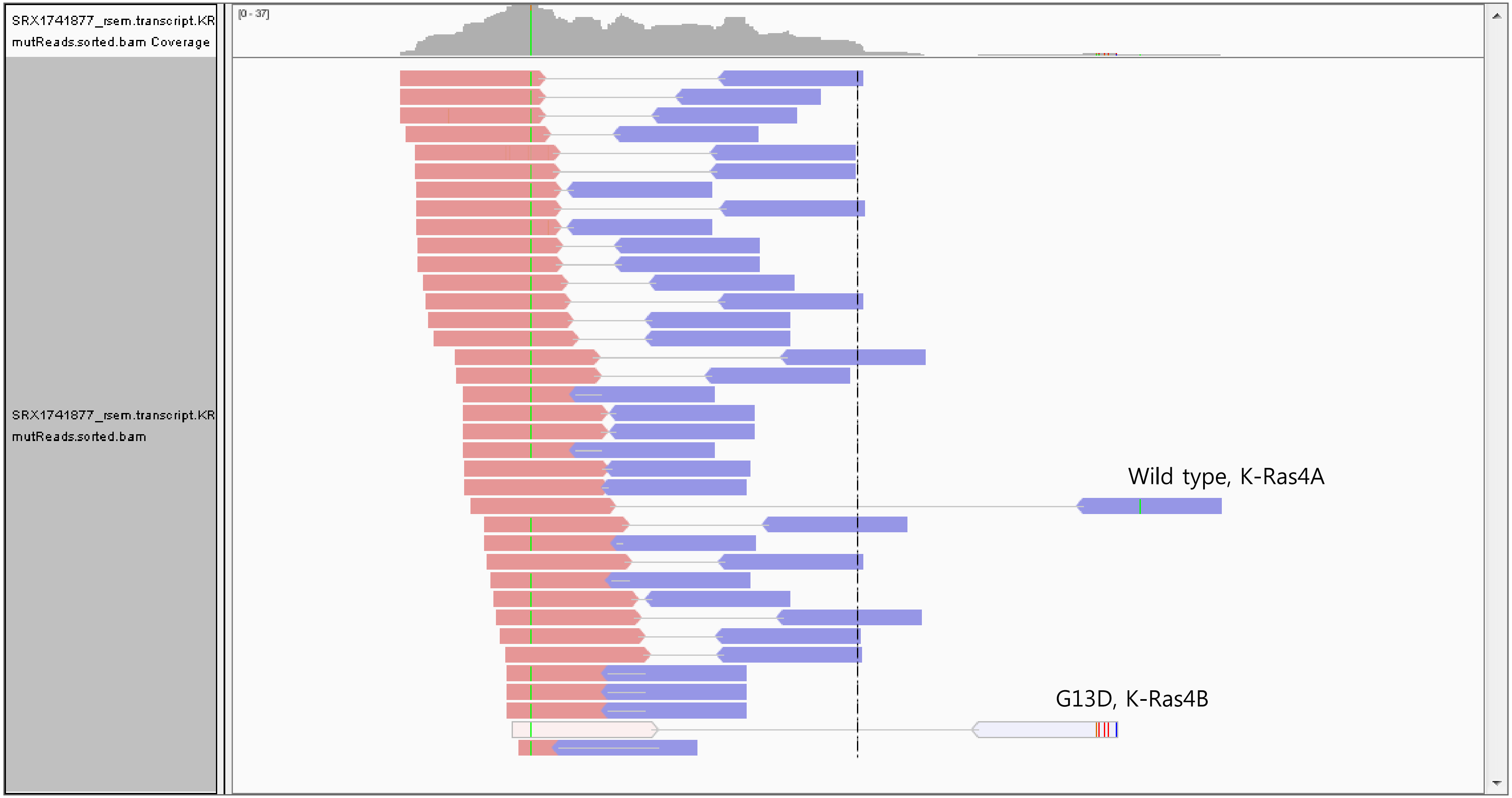

Only two read pairs were confirmed for both *KRAS* mutation status and isoform origin. The remained pairs were unknown for whether they were originated from K-Ras4A or K-Ras4B isoforms.

Figure S7 (continued)

C SRX1741936 (Q61H)

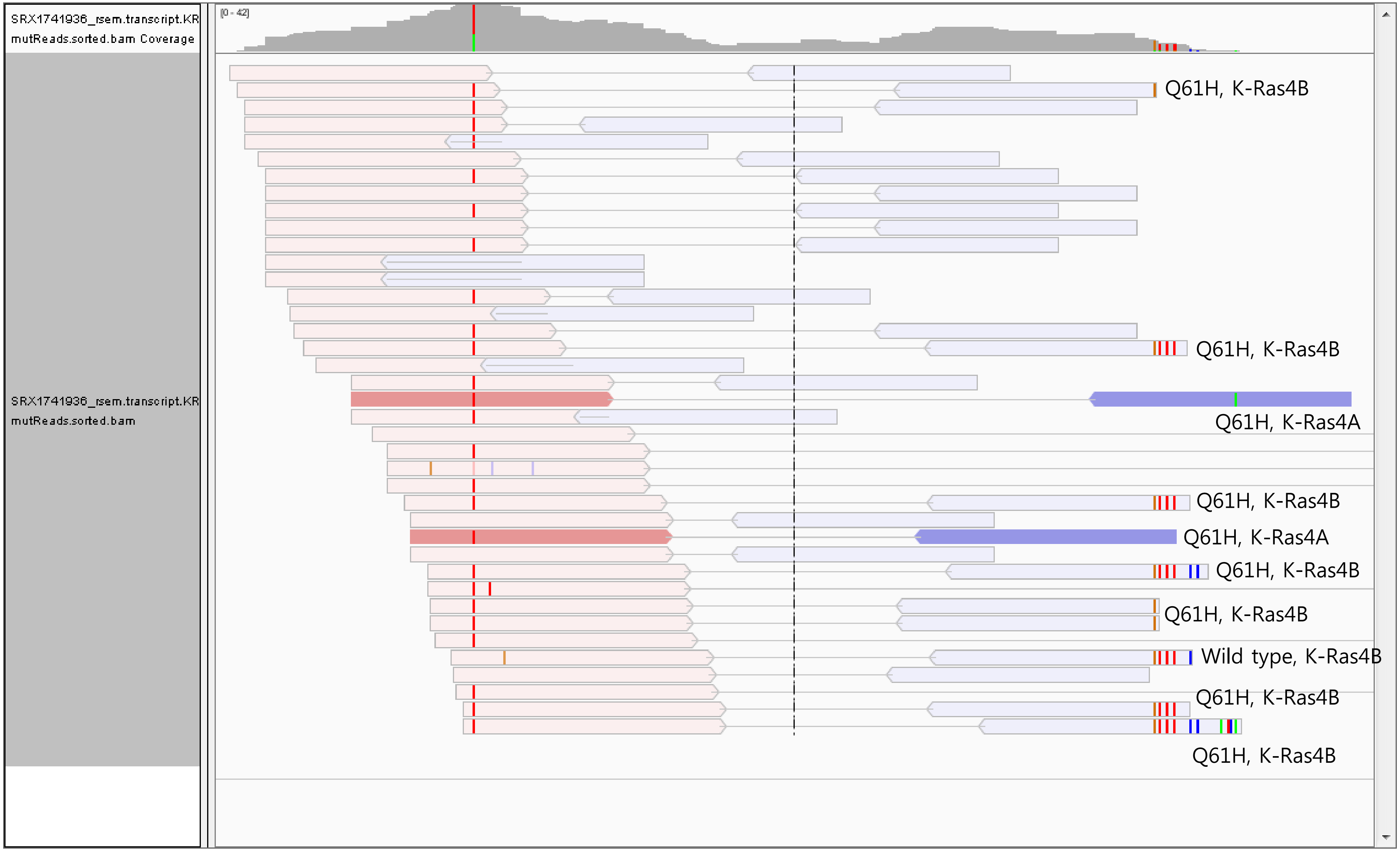

Eleven read pair was confirmed for both *KRAS* mutation status and isoform origin.
The remained pairs were unknown for whether they were originated from K-Ras4A or K-Ras4B isoforms.

Figure S7 (continued)
